# Polysaccharide-Based Systems for Targeted Stem Cell Differentiation and Bone Regeneration

**DOI:** 10.3390/biom9120840

**Published:** 2019-12-06

**Authors:** Markus Witzler, Dominik Büchner, Sarah Hani Shoushrah, Patrick Babczyk, Juliana Baranova, Steffen Witzleben, Edda Tobiasch, Margit Schulze

**Affiliations:** 1 Department of Natural Sciences, Bonn-Rhein-Sieg University of Applied Sciences, von-Liebig-Str. 20, 53359 Rheinbach, Germany; markus.witzler@h-brs.de (M.W.); dominik.buechner@h-brs.de (D.B.); sarah.shoushrah@h-brs.de (S.H.S.); patrick.babczyk@h-brs.de (P.B.); steffen.witzleben@h-brs.de (S.W.); edda.tobiasch@h-brs.de (E.T.); 2 Laboratory of Neurosciences, Department of Biochemistry, Institute of Chemistry–USP, University of São Paulo, Avenida Professor Lineu Prestes 748, Vila Universitaria, São Paulo, SP 05508-000, Brazil; jbaranova@usp.br

**Keywords:** polysaccharide, composites, bone tissue engineering, stem cells, osteogenesis, angiogenesis

## Abstract

Bone tissue engineering is an ever-changing, rapidly evolving, and highly interdisciplinary field of study, where scientists try to mimic natural bone structure as closely as possible in order to facilitate bone healing. New insights from cell biology, specifically from mesenchymal stem cell differentiation and signaling, lead to new approaches in bone regeneration. Novel scaffold and drug release materials based on polysaccharides gain increasing attention due to their wide availability and good biocompatibility to be used as hydrogels and/or hybrid components for drug release and tissue engineering. This article reviews the current state of the art, recent developments, and future perspectives in polysaccharide-based systems used for bone regeneration.

## 1. Introduction

Modern regenerative medicine has opened up a wide and multidisciplinary field of tissue engineering, where scientists try to mimic the function and properties of natural tissues. In the field of bone tissue engineering (BTE), scaffolds and stem cells can be combined to recapitulate the lost or repair the damaged bone tissue. Bone fractures, tumors, infections, and accidents occur, which require bone replacement. Small bone defects, such as minor fractures, heal slowly and result in the recapitulation of bone integrity. However, delayed unions, non-unions, and critical-sized bone defects, which cannot be healed naturally during the lifespan of the organism, require special regenerative medicine approaches. Currently, bone autografting is the gold standard of bone replacement, because autografts possess osteoinductivity, osseointegrity, osteoconductivity, and absolute compatibility. Unfortunately, the morbidities associated with bone autografting and scarcity of the material represent serious issues for a number of patients requiring bone replacement [1]. Novel alternative methods for bone regeneration are needed, which will eliminate the existing obstacles. Tissue-engineered scaffold-based grafts combining stem cells and bioactive molecules are emerging as a feasible alternative, and will likely replace bone autografts and acellular grafts in the future [2].

This article gives an overview on bone structure, metabolism, and repair, before reviewing recent advances in polysaccharide-based systems for bone regeneration, including pure polysaccharide systems and hybrid materials, their structure, chemistry, properties, and applications in osteogenesis, angiogenesis, and drug release.

## 2. Important Aspects of Bone Structure and Metabolism for Tissue Engineering

Bones ensure locomotion, stabilize, and protect the internal organs; support hematopoiesis; and the endocrine system, thus being crucial for the organism’s function. Bone is a rigid organ that consists of mineralized connective tissue, composed of an apatitic phase that makes up to 65% of its weight, and the remainder is made up of organic substances (mainly collagen type I) and water constitute [3,4,5].

Bone tissue undergoes growth, repair and renewal processes throughout life. Bone metabolism is mediated by anabolic osteoblasts, catabolic osteoclasts, and osteocyte signaling [6,7,8]. The interplay between bone-forming and bone-resorbing cells creates the unique equilibrium of bone tissue, which contributes to its strength, durability, and functionality. Bone resident, endothelial and bone marrow cells communicate to each other, regulating bone turnover and vascularization. Vasculature of bone tissue is vital for nutrients supply to the bone cells and bone marrow as well as for local and systemic signal and metabolite exchange [3,7,9,10].

### 2.1. Bone Cells and Their Role in Bone Metabolism

Osteoblasts, as cells involved in bone matrix formation and mineralization, compose 4–6% of bone cell population and reside along the bone surface. Osteoblasts originate from mesenchymal stem cells (MSCs) through stepwise differentiation process [11]. Early stages of MSCs differentiation towards osteoblasts are characterized by RUNX2 expression, which induces the expression of bone matrix protein genes such as alpha-1 type I collagen (COL1A1), alkaline phosphatase (ALP), bone sialoprotein (BSP) and osteocalcin (OCN) in the differentiating cells through the Wnt and bone morphogenetic proteins (BMPs) signaling [12]. Mature osteoblasts express Osterix and actively secrete bone matrix proteins such as OCN, BSP I/II, and COL1A1 [6]. After the synthesis of collagenous matrix, osteoblasts mediate hydroxyapatite deposition. Although some osteoblasts undergo apoptosis and others become entrapped in the mineralized matrix, an active pool is always maintained and is mobilized for bone remodeling and repair.

Bone-forming osteoblasts are partly entrapped inside the mineralized tissue and become osteocytes [6,13]. Osteocytes make up 90% of bone cells and have the longest life span [14]. They are individually encased in lacunae forming the lacunocanalicular system that connects the osteocytes to osteoblasts, bone lining cells, and the vascular supply [13]. Through this system, osteocytes modulate bone remodeling by controlling osteoblastic and osteoclastic activities. They also release factors (like osteoprotegerin, receptor activator of nuclear factor kappa-Β ligand (RANKL), and sclerostin) and hormones (like fibroblast growth factor 23) that exert effects on bone and other tissues [6,15]. 

Another population of osteoblast descendants, the bone lining cells, reside on the bone surface and can be mobilized to dedifferentiate into osteoblasts, thus comprising another pool of osteoblasts among hypertrophic chondrocytes from the cartilage, perivascular osteoprogenitors and bone marrow mesenchymal stem cells during adulthood [16,17]

Osteoclasts, multinucleated bone resorbing cells of hematopoietic origin, oppose the action of osteoblasts. They are formed via fusion of their precursors of monocyte–macrophage lineage, which are recruited to the bone from the circulation or the bone marrow. Under the influence of the macrophage colony-stimulating factor (M-CSF), which is secreted by mesenchymal stem cells and osteoblasts, and RANKL, secreted by osteoblasts; these precursor cells fuse and differentiate into mature osteoclasts [13,18,19]. Osteoclasts polarize and form the sealing zone and the ruffled border at the bone surface, where they pump the protons into the resorption lacuna thus lowering the local pH and facilitating the hydroxyapatite degradation [20]. They also secrete cathepsin K (CTSK) and matrix metalloproteinase, which degrades the collagenous matrix. [13,21,22].

### 2.2. Bone Remodeling and Fracture Healing

**Figure 1:**
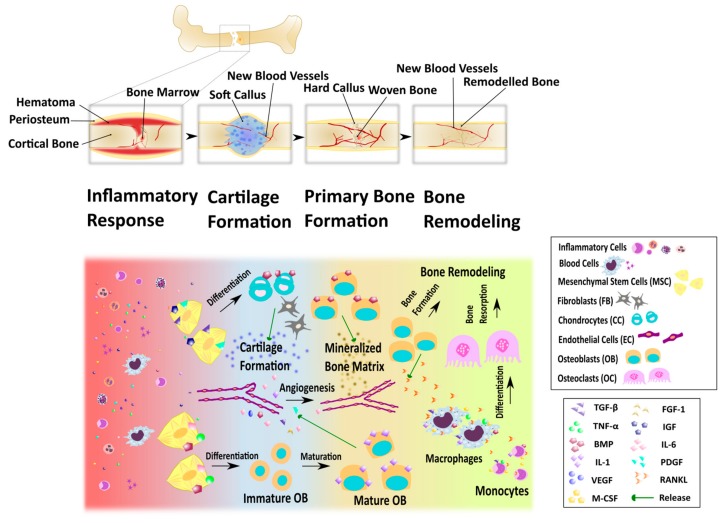
Stages of bone fracture healing. Upper part: changes that occur with the bone tissue during repair process; lower part: some of the cellular aspects and the factors that take part in the bone repair processes in each stage.

Native fracture repair is a complex process involving immune system activation, cellular migration, differentiation, and apoptosis. Fracture repair can be divided into four overlapping processes: inflammatory response, cartilage formation, primary bone formation, and bone remodeling (Figure 1) [23]. 

When a bone fracture occurs, the damage to the surrounding soft tissues and blood vessels causes a hematoma in this area and activates nonspecific wound healing pathways [23,24]. The proinflammatory response results in the release of cytokines and growth factors such as transforming growth factor β (TGF-β), fibroblast growth factor-2 (FGF-2), vascular endothelial growth factor (VEGF), M-CSF, interleukins such as 1 and 6 (IL-1 and -6), BMPs, platelet-derived growth factor (PDGF), and tumor necrosis factor α (TNF-α) by tissue and immune cells [24,25]. The release of TNF-α, IL-1, IL-6, IL-11 and IL-18 promotes angiogenesis [26,27], while TNF-α signaling induces osteogenic differentiation of MSCs [28].

The hematoma is replaced by a fibrovascular tissue [24] and mesenchymal stem cells are recruited to the site, where they differentiate into chondrocytes, produce cartilaginous matrix, and replace the fibrous tissue. Growth factors such as TGF-β, PDGF, FGF-1 and insulin-like growth factor (IGF) support fibroblast and chondrocyte proliferation and differentiation [23,24]. The stabilizing fibrocartilaginous plug or soft callus between the bone fragments is then formed and provides the platform for bone matrix formation [29]. TGF-β, PDGF, FGF-1, VEGF, and BMPs promote the invasion of the vascular endothelial cells into the callus [30].

Next, the removal of the soft callus and its replacement by a hard-bony callus, a process called endochondral ossification, occurs simultaneously with revascularization. Osteoblasts synthesize the initial woven bone matrix and mediate the mineralization, whereas growing vasculature supplies them with sufficient oxygen and nutrients [23,29]. 

The last stage of bone regeneration entails the remodeling of the woven bone hard callus into the mature bone. Irregular woven bone is gradually resorbed by osteoclasts and the lamellar bone is formed by osteoblasts, alongside with establishment of the vascular network [29].

### 2.3. Angiogenesis in Bone Transplants

**Figure 2:**
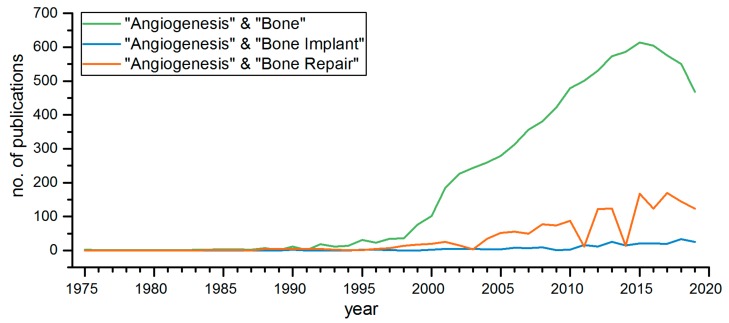
PubMed search for publications on angiogenesis and bone on 23 September 2019 with the following search terms; “angiogenesis & bone”, “angiogenesis & bone repair”, and “angiogenesis & bone implant”. The number of publications was stable until the late 1990s and rapidly increased during the 2000s.

Angiogenesis is the formation of new blood vessels within a tissue and is necessary to supply the cells with nutrients and oxygen. All bone cells as well as bone marrow require oxygen, nutrients and signaling molecules and therefore, a bone graft has to be well vascularized. Vascularization is especially important in large bone defect repair, where it plays a crucial role in local signaling transmission and provides a route for endogenous cells invasion into the graft, thus facilitating the healing and integration processes. Much of the research effort is focused on bone grafts vascularization, as the steadily increasing number of publications dealing with angiogenesis in bone and bone implants demonstrates (Figure 2).

To reach an optimum blood supply in grafts, different strategies have been used. As a first step, co-cultures with bone forming osteoblasts and endothelial cells, which form the inner layer of blood vessels, are usually cultivated together under the influence of different chemokines, to investigate the angiogenic potential. Inomata and Honda cultivated osteoblast-like MG-63 cells and human umbilical vein endothelial cells (HUVEC) on microfiber scaffolds, and showed that the endothelial cells were activated by the osteoblasts and that the osteogenic differentiation was enhanced on the scaffold [31]. The use of mesenchymal stem cells is also very common, as it is known that they have a paracrine effect on their environment. Piard and colleagues printed HUVECs and MSCs at different distances and showed that the indirect contact between both cell types prompt the release of paracrine signals, which stimulates angiogenesis and enhanced bone regeneration [32]. Another pro-angiogenic factor is hypoxia. The working group of Ma, for example, investigated the pre-vascularization of a tissue construct by co-culturing human osteoblasts and endothelial cells after hypoxic treatment. They suggest that hypoxia-induced angiogenesis is regulated by a complex balance of angiogenic and antiangiogenic factors and can be achieved by short-term repetition of hypoxia, but not long-term hypoxia [33]. Another interesting strategy is gen-cell therapy by transfection of MSC overexpressing BMP-2 and VEGF, which are important factors during osteogenesis and angiogenesis. The group of Lee recently showed that this strategy can induce rapid angiogenesis and osteogenesis in large bone defects [34]. Nevertheless, the most promising and commonly used strategy to produce a vascularized bone transplant is the use of a scaffold. Following, recent examples of vascularization studies using scaffolds are presented, while detailed information on scaffold materials are covered in a later section. 

Scaffolds can be either hard scaffolds or gels, which are seeded with the needed cell types or MSCs, which are differentiated towards osteoblasts and endothelial cells by using ligands or growth factors. Wang and colleagues seeded bone marrow MSCs (BM-MSC) onto nano-hydroxyapatite/collagen I/poly-l-lactic acid scaffolds and implanted them into rabbits with avascular necrosis of the femoral head [35]. The use of porous scaffolds and their comparison was done by the group of Murayama. They compared different types of porosity in β-tricalcium phosphate scaffolds (β-TCP), in rats and showed that the pore structures were filled with capillaries after three weeks [36]. The same kind of scaffolds was also used by Gu and colleagues. This group investigated the effect of magnesium ion concentrations on osteogenesis and angiogenesis of BM-MSC and HUVEC in 3D printed scaffolds in vitro [37]. Bone regeneration with membranes is a very new strategy, which was investigated by the group of Ye. They used PCL/chitosan/Sr-doped calcium phosphate electrospun nanocomposite membranes and showed an increased angiogenesis of BMSCs [38]. The use of extracellular vesicles (Exosomes) is also getting more popular. Tang and colleagues showed that exosomal MMP2 derived from mature osteoblasts promote angiogenesis of endothelial cells.

Beyond all mentioned approaches, the use of allografts in animal models is still common. This was recently shown in pigs by the working group of Houben, using bone vascularized composite allotransplants, and Wagner and colleagues who used allografts with adipose-derived MSC and showed an enhanced osteogenesis and angiogenesis depending on the optimized cell/volume ratio [39,40]. Wang first compared umbilical cord-derived MSCs (UC-MSC) to BM-MSC due to their angiogenic effect on HUVEC in vitro, and then showed a higher angiogenic potential of UC-MSC in hind limb ischemia in mice [41]. Nevertheless, the approaches using synthetic scaffolds seem to be the most promising ones in future use.

## 3. Scaffolds for Bone Tissue Engineering

Not all bone damages can be repaired via the natural bone regeneration pathway. Large defects would take more than a lifespan of a patient to heal naturally, thus bone grafts are currently the most used method to treat such defects [42]. Bone autografts are considered the gold standard treatment, mainly because of their immune and histological compatibility, but the need for an operation to acquire the graft and limited quantity of attainable material limits its use [43,44,45]. Allografts and xenografts possess high risks of immune rejection and disease transmission [43,46]. Scaffolds combined with appropriate cells are the other option to produce a compatible bone graft. If prepared in conjunction with the patients’ autologous cells, they have no risk of immune rejection or disease transmission [47]. Synthetic scaffolds can be prepared in sufficient quantities and shaped according to local requirements.

For creating scaffold-based bone grafts, cells with good proliferative and osteogenic capacity are needed. Mesenchymal stem cells present in various niches in the adult body, such as bone marrow, adipose tissue, dental pulp, and umbilical tissues, show promising results in preclinical models and thus are considered as a potential cellular source for bone tissue regeneration and repair of large bone defects [43,47,48], but their lifespan is limited and differentiation efficacy is decreasing with patients age [42,49]. A promising approach to counter this issue is to preselect the MSCs according to their osteogenic potential or modulate their properties via activating certain cell signaling pathways. For example, modulating purinergic receptors with specific ligands can enhance the integration and vascularization of the graft [43,50]. Scaffolds can also be combined with growth factors to enhance their osteoinductivity, osteoconductivity, and induce angiogenesis. Some of these factors are already being used in clinical trials. Examples of such factors are BMP-2 and BMP-7 that, after being tested in animal models and clinical trials, now have been FDA approved. VEGF is another possible choice of growth factors, since it plays a significant role in angiogenesis and osteogenesis, and is already being tested in animal models [51,52].

Besides the appropriate source for bone cells and adequate vasculature, scaffolds are important for supporting cell functions, as they provide a platform for cell adhesion, migration, three-dimensional spatial arrangement, and serve as crystallization centers for hydroxyapatite. The ideal scaffold-based graft should mimic or induce the formation of the native bone matrix, which accommodates the bone producing and resorbing cells as well as blood vessels. It further contains various growth factors, cytokines, and osteoinductive and angiogenic signaling molecules [53,54]. The graft must also be easily integrated into the host tissues and have an adequate resorption profile. Careful evaluation of the graft material disintegration, bioactive compound release, and resulting metabolites are necessary to avoid cytotoxicity and impairment of the grafting process with resulting harm to the host [42,55]. The integrated, cellularized bone graft should be fit to bear the pressure and tension, which is prominently exerted on the bones of the limbs, spine, and chest, without compromising or limiting the host performance. 

There are numerous ways and strategies in how to achieve and fabricate bone tissue scaffolds, including the use of metals, polymers, ceramics, and hybrid systems, all having their own advantages and challenges [56]. Metals are one of the oldest materials used in bone surgery and widely used as replacement and fixation implants. Despite having good biocompatibility and superior mechanical strength, they often have to be removed in another surgical procedure due to their non-biodegradable properties. This is why current research focuses on biodegradable scaffold materials made of polymers, ceramics, and hybrids, often combining them to overcome their individual disadvantages [57]. The used polymers can be synthetic (such as poly(ε-caprolactone), poly(lactic acid), or polyurethanes) or naturally sourced (such as collagen, polysaccharides, or lignin-based). These polymers are usually biocompatible, flexible, biodegradable, and can be easily modified, but they have poor mechanical strength [58]. Ceramics often consist of calcium phosphates (such as hydroxyapatite (HA, Ca_10_(PO_4_)_6_(OH)_2_), tricalcium phosphates (TCP, Ca_3_(PO_4_)_2_)) or bioactive glasses. They are biocompatible, can support cell adhesion and differentiation, but are brittle and may have a slow degradation rate [59,60,61]. Hybrid materials comprised of both, polymers and ceramics, combine their advantages, resulting in biocompatible, cell supporting systems that have reasonable mechanical properties. They often aim to mimic natural bone, which itself is comprised of water; organic components (mainly collagen type I and other proteins), providing flexibility; and mineral components (such as calcium phosphates), providing toughness and strength [53,62,63,64,65,66]. Polymeric, ceramic, and hybrid systems are also used in drug and growth factor delivery, since they can be tuned in order to release substances in a controlled way. However, the extent of sustaining drug release always depends on the individual drug, the material, the release medium used, and the desired effect the drug or factor should have [51,65,67,68].

Recent developments towards sustainable materials also changed the focus in bone tissue engineering from traditional polymers to those from natural sources. Here, polysaccharides are one of the most promising materials due to their wide availability, biocompatibility, easy handling, and modification. 

### 3.1. Polysaccharide Scaffolds

**Figure 3:**
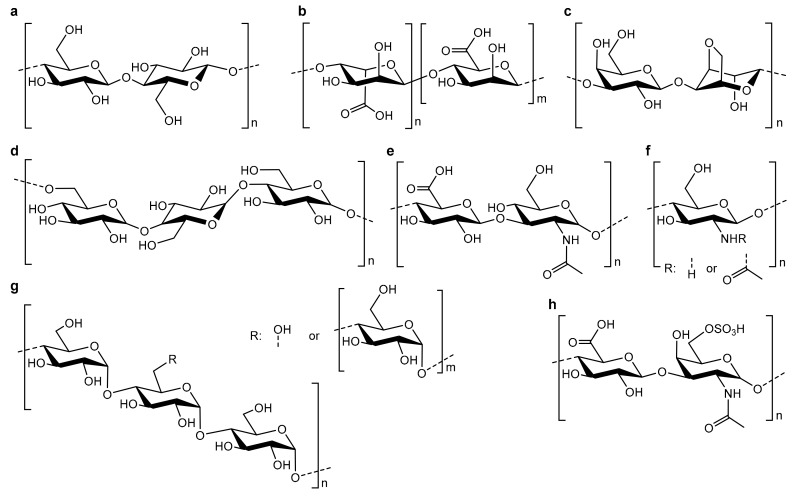
Overview of the most common polysaccharides used for bone tissue engineering applications: (**a**) cellulose, (**b**) alginate, (**c**) agarose, (**d**) pullulan, (**e**) hyaluronic acid, (**f**) chitosan, (**g**) starch, and (**h**) chondrotin-6-sulfate.

Polysaccharides are widely used in bone tissue engineering, often as hybrid materials in combination with bioceramics, but also as pure polymeric systems. The most common polysaccharides (Figure 3) used are alginate, hyaluronic acid, chitosan, and starch, but agarose, other glucans, dextran, and pullulan also find their application in several studies. Usually, the unmodified polymer is used for hydrogel or capsule formation, but in some cases, derivatization of the polysaccharide is performed to tune its functionality [69,70,71]. 

The advantages of polysaccharides are their availability, their good biocompatibility, and usually even biodegradability, and that they provide cell attachment and growth. On the other hand, being of natural origin, they may cause immune responses and often have only poor mechanical properties [72]. Hence, even in pure polymer systems, polysaccharides are often used in conjunction with other (natural) polymers and/or are cross-linked or modified in order to achieve a material more suitable for bone tissue engineering.

Alginate is one of the most commonly used polysaccharide, as it is easily cross-linked with multivalent cations such as Ca^2+^. It consists of blocks of β-d-mannuronic acid (M) and α-l-guluronic acid (G) linked via (1→4) bonds. A higher ratio of G blocks to M blocks facilitates gelling and results in stronger, more stable hydrogels. The anionic alginate can be extracted from seaweeds commercially in high purity and has excellent biocompatibility. In tissue engineering, alginate is mainly used as beads for the controlled release of drugs or growth factors, or as encapsulation medium for cells. For example, alginate scaffolds made by freeze-casting and cross-linking with CaCl_2_ were loaded with bone forming protein-1 (BFP-1) and showed improved cell-adhesion and osteogenesis behavior in vitro and in vivo. BFP-1 was released continuously over 21 days, human osteoblast-like MG-63 cells showed higher proliferation rates and alkaline phosphatase (ALP) activity on scaffolds loaded with low and medium levels of BFP-1, while too high levels of BFP-1 restrained proliferation. In vivo tests on beagle dogs showed improved bone formation in calvarial defects [73]. Alginate was used together with TEMPO-oxidized cellulose nanofibrils in a 3D-printing process to obtain scaffolds with a defined macroporous structure. The hydrogel scaffolds were subjected to chemical characterization, mineralization studies with simulated body fluid (SBF), and mechanical testing. The best printable composition was found to be 50% alginate/50% nanofibrils and printed scaffolds were cross-linked by soaking in CaCl_2_. Compressive strength increased with the addition of nanofibrils and scaffolds exhibited hydroxyapatite mineralization in SBF with crystallite sizes of around 25 nm [74]. The suitability of cross-linked alginate scaffolds compared to collagen scaffolds was evaluated using human mesenchymal stem cells (hMSC), human umbilical vein endothelial cells (HUVEC) and RNA determination in vitro. Here, alginate scaffolds proved to be beneficial for osteogenesis, however, collagen scaffolds were superior for simultaneous vascularization of bone in hMSC/HUVEC co-culture [75]. Alginate scaffolds aerogels were prepared using pressurized CO_2_ for foaming and simultaneous gelation from CaCO_3_. Lignin, a major component of wood and grasses and a by-product of the pulping industry, was also incorporated into these scaffolds by simple mixing. Scaffolds with pore sizes of 200 to 450 µm showed good cell viability in both indirect and direct MTT assays [76]. Beads of 1 mm diameter made from alginate and a xyloglucan extracted from tamarind seeds were cross-linked with calcium ions and loaded with the anti-diabetic drug metformin, which showed a zero order release profile under both pH 2 and pH 7.4 [77]. A more complex system of alginate, gelatin, and hyaluronic acid films cross-linked with EDC and incorporated bone ash showed good mechanical stability and biomedical applications. Release of 5-fluorouracil was found to be pH-dependent with increasing release rate at higher pH [78].

Starch, a natural mixture of amylose (linear α-(1→4)-linked d-glucose) and amylopectin (additional α-(1→6)-linkages), is used for its ability to form gels. Hydrogels made from unmodified corn starch, lignin, or hemicelluloses, each cross-linked with citric acid via reactive extrusion, were investigated regarding their mechanical properties and swelling behavior. The starch-based scaffold showed the highest swelling, but also has the highest compression modulus, likely due to the much higher molecular weight of starch compared to lignin and the hemicelluloses used. The process seemed promising; however, further studies regarding biocompatibility or cell interaction have not yet been published [79]. Starch aerogel microspheres loaded with ketoprofen drug were incorporated into poly(ϵ-caprolactone) (PCL) scaffolds made by supercritical CO_2_ foaming. These highly porous scaffolds showed good mechanical properties, water permeability, cell infiltration capacity, and ketoprofen release in vitro over 3 days [80].

Hyaluronic acid is a linear glucosaminoglucan (comprised of d-glucuronic acid and *N*-acetyl-d-glucosamine, linked with alternating β-(1→4) and β-(1→3) glycosidic bonds) that is a major part of human extracellular matrix. With its biocompatibility, enzymatic degradability in vivo, and mechanical properties, it is a good candidate for the use in regenerative medicine. However, due to its water-solubility, it has to modified and/or used together with other materials. Hydrogels made by cross-linking maleimide-functionalized hyaluronic acid with a cell-adhesive and a degradable peptide, subsequently loaded with BMP-2 and SDF-1a showed a release of these growth factors that correlated strongly with the degradation of the scaffold [81]. Another system used hyaluronic acid and aminocellulose for layer-by-layer coating of poly(methyl vinyl ether/maleic acid) nanoparticles. These layered particles showed antibacterial and antimicrobial properties, and proved to be non-toxic to human fibroblasts [82]. An injectable BMSCs-laden hydrogel system comprised of modified hyaluronic acid and modified chondroitin-sulfate has recently been reported. The polysaccharides were grafted with tyramine and subsequently cross-linked with H_2_O_2_ and horseradish peroxidase. The prepared scaffolds were loaded with bone marrow-derived rat BMSC and BMP-2. BMSC seeded on the scaffold showed increased proliferation, osteogenic differentiation, and ALP activity after 11 days of cell culture in osteogenic medium. Several osteogenic markers (including ALP, Col I, RunX2, and OCN) were also evaluated via RT-PCR after 14 days and showed increased expression. In vivo studies on rats showed improved bone healing and regeneration after 4 weeks [83]. Although clinical studies on novel materials are rare, one uses a hyaluronic acid-based bone graft for sinus augmentation. The study compares the material to commonly used collagenated heterologous bone and finds enhanced bone formation after 4 months [84]. Additional to their use in bone tissue regeneration, systems with hyaluronic acid could also play a role as release systems for drugs or growth factors.

One of the most commonly used polysaccharides is chitosan, a glucosamine composed of β-(1→4)-linked d-glucosamine and *N*-acetyl-d-glucosamine units. It is obtained through deacetylation of chitin, with the degree of deacetylation being responsible for the chitosan’s properties. It can act as a polycation due to its deacetylated amino groups and support cell attachment, differentiation, and migration, as well as osteoconduction and the promotion of osteoblast growing, making it a useful material for bone regeneration. However, residues of proteins may cause immunoreactions. Quaternized chitosan grafted with polyaniline was cross-linked with oxidized dextran in order to obtain an injectable and in vivo-setting hydrogel. It proved to have antibacterial properties and stimulated C2C12 myoblast formation. Rabbit adipose tissue-derived MSC showed good survival and proliferation rates on the scaffolds [85]. Very recently, scaffolds based on chitosan and gelatin have been synthesized for use with dental pulp stem cell (DPSC) differentiation and alveolar bone regeneration. In vitro tests with DPSC showed no cytotoxic effects and scaffold mineralization over 8 weeks. In vivo experiments in mice confirmed high amounts of osteoid and fully mineralized bone [86].

Another widely used polysaccharide is agarose. The polymer consists of repeating disaccharide blocks of (1→4)-linked β-d-galactose and 3,6-anhydro-α-l-galactose, linked via (1→3) glycosidic bonds and is extracted from red algae. It is biocompatible, non-cytotoxic, and forms thermoreversible hydrogels, making its application very easy [87]. However, it has poor cell attachment properties, which is why it is often used in combination with other polymers or in hybrid systems. A blend of agarose and collagen type I was optimized for 3D bioprinting purposes. Human umbilical artery smooth muscle cells (HUASMCs) were encapsulated in the gel and printed into scaffolds. This resulted in stable gels with viable cells; however, further tests and improvements are still needed [88]. Another pure-polymer system is a hydrogel of agarose and lignin, cross-linked with epichlorohydrin. Gel strength and stiffness increase with increasing amount of agarose, lignin and cross-linker. However, no application of these gels for tissue engineering has been reported yet [89]. As already mentioned, agarose is often used together with ceramics to get reinforced gels that also support cell adhesion.

Cellulose, a polymer of (1→4)-linked β-d-glucose units, and its derivatives, as well as other glucose-derived polysaccharides such as pullulan, dextrans, and glucans, are also used in bone tissue engineering [90]. Recently, nanocellulose with incorporated sulfate and phosphate groups was cross-linked with hydrazone and cast and dried into aerogels. In vitro evaluation of Saos-2 cells on these porous scaffolds showed increased cell metabolism. Scaffolds implanted into Long Evans rats showed increased bone volume and osteoconductivity after 12 weeks [91]. Electrospun scaffolds consisting of cellulose acetate were evaluated regarding their in vitro degradation behavior, cytotoxicity on L929 cells and their application as release platform for dexamethasone. Results show a degradation of less than 25% in 150 days and no adverse effects on L929 fibroblasts. The scaffolds exhibit a biphasic release behavior into PBS, with an initial burst release of less than 20% and prolonged release over 180 days [92]. Pullulan and human-like collagen were cross-linked with diepoxyoctane, washed, cast into hydrogels, and subsequently freeze-dried. These scaffolds were then subjected to degradation, cytotoxicity, cell viability and hemolysis tests as well as cell attachment and in vivo performance analysis in New Zealand rabbits. Degradation studies using pullulanase showed a degradation of up to 25% within 8 weeks. Cell viability ranged between 80 and 90% for most of the formulations; hemolysis and cytotoxicity tests showed no adverse effects. In vivo studies revealed less inflammation and better vascularization of cross-linked pullulan-collagen scaffolds after 8 weeks compared to pure cross-linked pullulan scaffolds [93]. Pullulan and dextran were dissolved in aqueous medium, cast into gels and cross-linked with sodium trimetaphosphate at 50 °C. Freeze-dried discs were used for cell seeding experiments, which resulted in homogenous cell distribution of viable MC3T3-E1 cells [94]. A different study compared a hydrogel made from pullulan and dextran cross-linked with sodium trimetaphosphate to a commercial biphasic calcium phosphate (HA/TCP). Rat bone marrow MSC seeded on the scaffolds attached well onto the ceramic and infiltrated the porous hydrogel in vitro. MSC-seeded scaffolds were also tested in vivo on Lewis rat femur cavities for up to 90 days. Results suggested different bone repair mechanisms of both types of scaffold. Hydrogels were resorbed very fast, were able to deliver growth factors and MSCs, and showed higher vascularization of the defect site than the ceramic scaffolds [95]. 

Tamarind seed powder contains xyloglucans and was used together with acrylic acid in a radical polymerization to form a stiff hydrogel. The material showed biocompatibility for HUVECs, cell adhesion and proliferation of bone-marrow derived preosteoclasts, and enhanced mineralization of MC3T3-E1 preosteoblasts. Murine MSC exhibited a higher proliferation on the scaffolds, even without osteoblast differentiation medium, making them potential candidates for biomedical applications [96].

### 3.2. Polysaccharide-Based Hybrid Scaffolds

#### 3.2.1. Calcium Phosphate Hybrids

Natural bone tissue is a composite material consisting of roughly 30% organic matrix (mainly collagen type I) and 65% calcium phosphate based bone mineral, which improves the mechanically poor properties of the organic matrix, resulting in the flexible but also tough mechanical behavior of natural bone. Chemically, bone mineral can be described as a hydroxyapatite phase containing multiple substituting ions such as sodium, magnesium, potassium, strontium, zinc, carbonate, silicate, chloride, and fluoride. Resembling the natural bone mineral, materials based on hydroxyapatite and other CaP-phases (Table 1) are extensively examined for the use in BTE, and examples have been in clinical use for decades [42,97,98,99]. To overcome the drawbacks of ceramic scaffolds for bone regeneration (namely mechanical brittleness, poor resorption, and missing vascular ingrowth), one main aspect of biomaterial science is the development of hybrid systems together with polymeric matrices. Here, the natural bone tissue is resembled more precisely and the lacking biological properties of single-component polymeric materials are overcome by adding bioactive CaPs.

Because of their bioactive properties, chitosan-based composite materials have been studied for use in BTE for a long time. Previous research in this field has been reviewed by a number of authors [100,101,102]. Recently, Chen et al. evaluated the effect of different CaP-Phases (namely CDHA, TCP & BCP as a mixture of both) on the bone regeneration activity of Chitosan/CaP-membranes in vitro and in vivo. The results indicate that CDHA adsorbs cell-adhesive proteins, and consequently primary osteoblast cells show increased adhesion and differentiation, while TCP provides an environment rich in calcium- and phosphate-ions through its enhanced solubility and thereby enhancing proliferation. By using BCP as an additive in the PS-matrix, the positive effects of the single phases are combined. Thus, in an in vivo test using critical-sized calvarial bone defects in rats, BCP-loaded membranes show the most effective bone regeneration, generating up to 57% newly formed bone in the original bone defect area after three weeks [103]. Zhou et al. compared the performance of hydroxyapatite and whitlockite (WH), a magnesium-containing CaP incorporated in chitosan membranes in vitro and in vivo. Chitosan/WH-composites show significantly elevated results for proliferation, ALP activity and the expression of osteogenic marker genes using human bone mesenchymal stem cells. These in vitro results are confirmed in a rat calvarial defect model, where the WH containing scaffolds show significantly higher values for newly formed bone and bone mineral density. The improved results for WH are mainly attributed to the presence and gradual release of Mg by the authors, which is in accordance with previous studies [104].

Recent publications on chitosan-based composite materials often deal with more complex material systems, e.g., by the addition of further matrix materials (Chondroitinsulfate [105], Glucan [106,107], Alginate [108]) or the use of novel/different cross linking agents replacing the established glutaraldehyde by e.g., vanillin [109] or dopamine [110]. Fan et al. combined the cross-linking of a chitosan-chondroitinsulfate matrix by EDC/NHS-chemistry with in situ synthesis of hydroxyapatite. In vitro tests show that the ternary system provides cytocompatibility and proliferation of osteoblast cells while enhancing the ALP-expression in comparison to the binary system without HA [105]. In a study conducted by Kim et al., alginate is used as a bio-inspired dispersant for HA-particles in combination with a chitosan-matrix. Besides improved mechanical properties and uniform pore structures, the osteoblastic differentiation of preosteoblastic MC3T3-E1-cells is also improved by addition of HA to the scaffolds as determined by ALP-activity and alizarin-red staining [108]. Przekora et al. modified the chitosan matrix of a HA-composite with β-1,3-Glucan and thereby enhancing the polarity due to the additional OH-groups. This results in a significantly higher adsorption of adhesion proteins that are crucial for osteoblasts [106]. In a later study, the compatibility of the chitosan-glucan-HA-composite with adipose tissue derived stem cells (ATSCs) and bone marrow derived stem cells (BMSCs) was evaluated. The results showed that both cell types proliferated and differentiated on the scaffold surfaces, with better results for BMSCs [107]. Injectable chitosan/HA-microspheres with varying content of HA were produced by Cai et al. by an emulsion technique and subsequent cross-linking with vanillin. In vitro assays on MG-63 human osteosarcoma cells revealed, that all formulations showed good results regarding cytocompatibility, cell adhesion and proliferation. However, best results were achieved with 29.5 wt.-% HA, which was the highest content in the series of tested materials [109]. By using dopamine as cross-linking agent, Prajatelista et al. established an anisotropic growth of needle-shaped HA-particles in chitosan membranes, resulting in a more homogeneous distribution, size, and shape of HA-particles compared to CS/HA-membranes without dopamine. This more ordered structure, however, did not result in improved biological properties, since in vitro tests with MC-3T3 preosteoblastic cells showed slightly lower results for cell viability and proliferation on dopamine-containing membranes than on non-cross-linked CS/HA-membranes [110].

Excellent biocompatibility and multiple readily available processing techniques make alginate a frequently used matrix material in hybrid scaffold materials together with CaPs. The effect of HA content on bone regeneration has been studied by Barros et al., incorporating 30, 50, and 70% of HA in spherical hydrogel beads, respectively. Beads with 30 % HA enhance the proliferation and osteogenic activation of human osteoblastic cells in vitro. In an ex vivo test, the 30% formulation also enhanced collagenous deposition and trabecular bone formation. In contrast, concentrations of 50 and 70 % HA showed less pronounced results, what the authors originate in the inhibitory effect of larger concentrations of dissolved Ca^2+^ on the activity of cells [111]. Jo et al. added silk fibroin to the alginate matrix of composite beads and compared their biological response in vitro and in vivo with alginate and alginate/HA-beads. Interestingly, alginate/HA-beads did not show better results in a MTT-test using MG63-osteoblast like cells than single-phasic alginate-beads, whereas the ternary system with silk fibroin shows superior cell growth after 48 and 72 h. Higher new bone formation and better biodegradability with no signs of inflammatory reaction together with high expression of osteogenic markers (FGF-23, OPG, and Runx2) of the alginate/silk fibroin/HA-system in an in vivo rat calvarial defect model supported these in vitro findings [112]. Alginate-fibrin beads encapsulating a calcium phosphate cement paste and induced pluripotent stem cell-derived mesenchymal stem cells (iPSMSCs) led to an injectable, self-setting bone filler that led to increased new bone formation in double cranial bone defects in rats after 12 weeks. Additionally, BMSCs were co-cultured with the microbeads showing higher expression of markers for osteogenic differentiation [113]. Factorial experimental design has been used by Nabavinia et al. to determine the optimum composition of alginate/gelatin/HA-microcapsules. The presence of HA has a major effect on cellular response as evidenced by proliferation and differentiation studies on encapsulated MG63 cells [114]. Porous alginate scaffolds are produced using 3D printing and subsequent coating with HA in an in situ mineralization process. By forming a thin layer of needle-like HA on the scaffolds surface, the in situ mineralization enables better adhesion, spreading and osteogenic differentiation of hBMSCs compared with a hybrid scaffold prepared by simple mixing of HA and organic matrix. Furthermore, the HA-coated scaffolds show a more sustained release of bovine serum albumin (BSA) compared to pure alginate- and mixed alginate/HA-scaffolds [115]. In a later study, the authors added gelatin to the HA-coated alginate scaffold-system. In vitro results with rat bone marrow stem cells (rBMSCs) confirm the earlier results, as the HA-coated samples stimulated the proliferation and osteogenic differentiation and enhanced the protein adsorption [116]. Grafts comprised of alginate combined with a strontium-containing carbonated HA were investigated regarding their biocompatibility on MC3T3-E1 cells in vitro. Samples with strontium performed better than those without, although both systems could be regarded as noncytotoxic. In vivo studies performing sinus lift procedures in rabbits showed similar performance of Sr-containing grafts compared to those without Sr with respect to biocompatibility, osteoconduction, and bone volume density after 4 and 12 weeks [117].

Biocompatible properties, facile handling due to thermoreversible hydrogel formation as well as high availability make agarose a promising matrix material for BTE applications. Incorporation of a CaP-phase via in situ precipitation leads to a homogeneous distribution of nanosized domains of the mineral in the organic matrix. In vitro -tests with MG-63 osteoblast like cells show good results regarding proliferation and enhanced ALP-activity as well as Ca-deposition by in situ-mineralized agarose-scaffolds compared to blank polysaccharide samples [118]. In a further study, the authors used carboxylated agarose and added silk fibroin to the material. Using in vitro studies, a dose-dependent positive effect of silk fibroin on MG63 cell adhesion, spreading, viability, proliferation and ALP-activity was observed and confirmed by an in vivo rat tibia defect model [119]. Recently, Witzler et al. published studies on agarose/HA composite scaffolds prepared via in situ mineralization. Along with no cytotoxicity on MG-63 human osteosarcoma cells and human adipose tissue derived mesenchymal stem cells, respectively, hybrid materials delay the release of ATP and suramin as model substances for osteogenic P2-receptor ligands for up to four days [120]. Paris et al. combined simple mixing of carbonated HA into an agarose solution with a patented shaping method (GELPOR3D^®^) to evaluate dual drug release properties of the composite scaffolds. Due to the unique affinity between bisphosphonate compounds and HA, the release of zoledronic acid is strongly sustained. In contrast, ibuprofen as an anti-inflammatory drug shows a burst release within three hours. Applying an additional encapsulation of ibuprofen in chitosan microspheres leads to a delayed release of more than two days [121]. In a more recent publication, the same authors report the modification of the described agarose/HA-composite by adding vascular endothelial growth factor (VEGF) and mesoporous silica nanoparticles loaded with the antibiotic cephalexin to improve vascularization and reduce infections during bone defect healing. Drug release studies, in vitro evaluation using MC3T3-E1 preosteoblastic cells and an ex ovo test for angiogenetic potential revealed a controlled cephalexin release by using either free or encapsulated drug-amounts, positive results for cell adhesion and cytotoxicity as well as the capacity to induce the formation of new blood vessels due to the release of VEGF [122]. To resemble the bone mineral more properly in a synthetic bone graft, Kazimierczak et al. prepared Zn- and Mg-doped HA and applied it with a foamed agarose/chitosan-matrix. The results of in vitro studies using MC3T3-E1 preosteoblastic cells, human bone marrow-derived stem cells, and human adipose tissue-derived stem cells demonstrate that Mg-incorporation increases spreading and proliferation of osteoblasts. However, osteogenic differentiation of MSCs is retarded by the incorporation of the dopant cations, since higher levels of osteogenic markers and mineralization activity are found for scaffolds with stoichiometric HA. This finding is surprising, as a stimulatory effect of Mg and Zn on osteogenic differentiation has been shown by numerous studies on substituted HA for BTE [123]. In a following study, the same authors investigated the influence of the solvent and concentration of NaHCO_3_ to be applied as foaming agent on the properties of agarose/chitosan/HA composite scaffolds. Using 2% acetic acid and 2% NaHCO_3_, uniform, interconnected networks of high porosity could be prepared. In vitro tests with MC3T3-E1 preosteoblasts showed positive results for cytotoxicity and favored cell adhesion as well as bioactivity in simulated body fluid [124]. Agarose microspheres loaded with BCP are prepared by a one-step “water in oil” method by Hasan et al. and their biological response is positively evaluated on MC3T3-E1 mouse pre-osteoblasts in vitro and on rats in vivo. However, by applying calcium sulfate dihydrate (CSD) as an additional additive, results of the binary agarose/BCP-system are significantly overcome in both, in vitro and in vivo assays due to the greater solubility of CSD and subsequently higher nutrition of involved cells with calcium ions [125]. N-doped graphene (NG) has been combined with HA in a hydrothermal synthetic process by Luo et al. and the resulting NG-HA is subsequently added to an agarose matrix. Besides improved mechanical properties, the combination of agarose, NG and HA in the scaffold material promotes adhesion, proliferation, and osteogenic differentiation of rat MSC compared to plain agarose and composites of agarose/HA and NG/HA. These promising in vitro findings are confirmed in vivo by the curing of a rabbit femur defect within 12 weeks [116]. 

As one of the most abundant polysaccharides, cellulose may be derived from either plants or bacteria and is investigated together with its derivatives like carboxymethylcellulose (CMC) as matrix material due to its inherent biocompatibility and biodegradability [126]. Combinations with different CaPs like HA [127,128,129,130], CDHA [131], CPC [132], and BCP [133,134,135] are recently evaluated for bone tissue regeneration.

**Figure 4:**
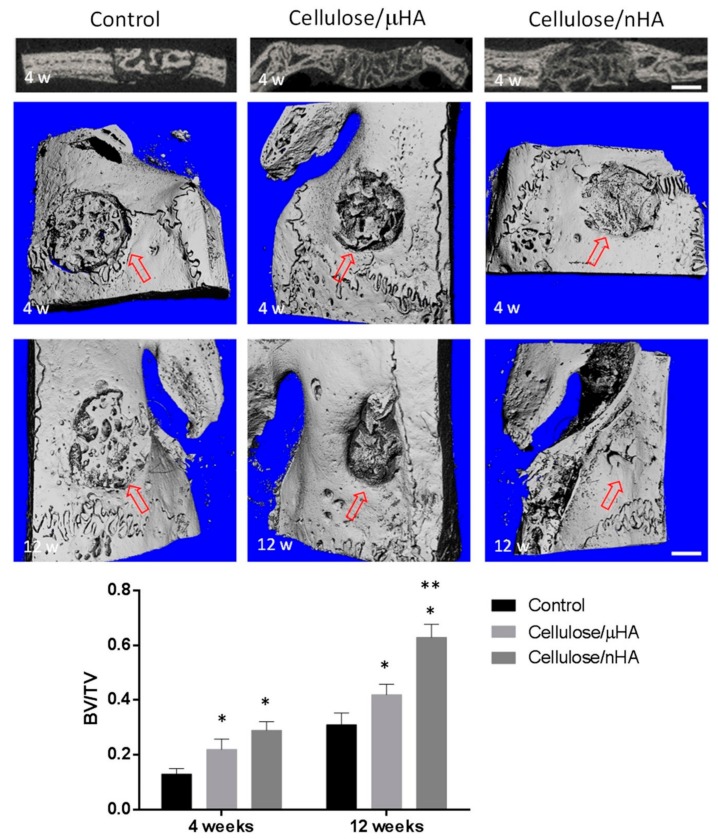
Results of an in vivo rabbit calvarial defect model treated with cellulose-based composites, using HA-particles with either 20 µm (µHA) or 100 nm (nHA) in diameter, compared to untreated control. Figures above: 2D images and 3D reconstructions by means of µ-CT of the control and implanted composites after 4- and 12-week treatment, respectively. Margin of the initially created defect is highlighted by red arrows. Scale bar corresponds to 3 mm. Graph below: Ratio of bone volume (BV) to total volume of the created defect (TV). * indicate a significant deviation from control; ** indicate a significant deviation from cellulose/μHA. Reproduced from work in [127] with permission from John Wiley and Sons, 2019.

**Figure 5:**
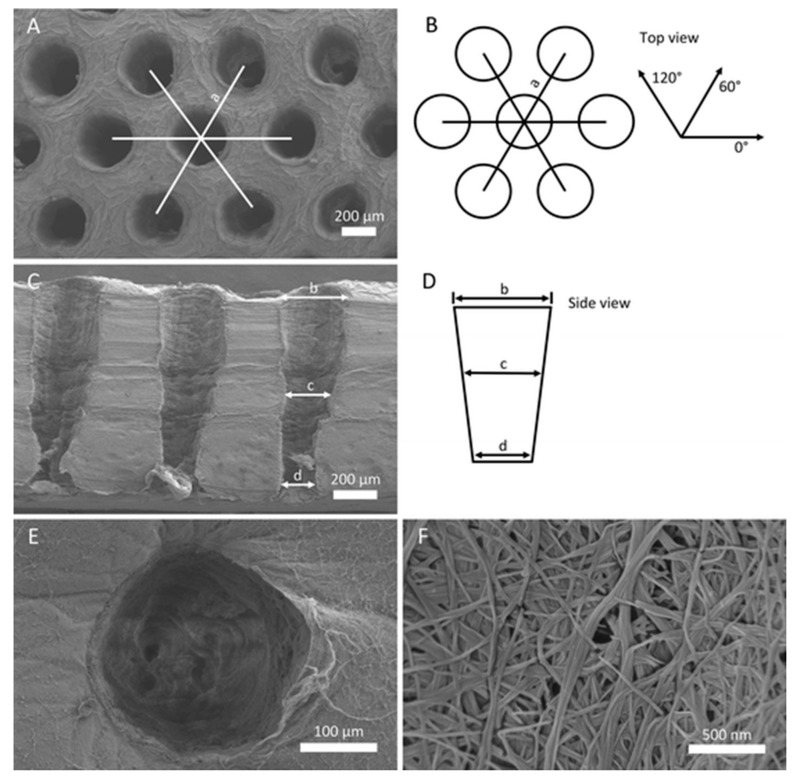
Defined porous structure of a nanofibrous BC-sheet scaffold prepared by laser perforation (**A**,**B**). Honeycomb arrangement of pores of trapezoid-shaped in cross section (**C**,**D**). Well-defined round pores of 300 µm in diameter (**E**) perforate the fibrous nanostructure of the BC-sheet scaffold (**F**). Reproduced from work in [128] with permission from Springer Nature, 2019.

The size of HA particles incorporated in a cellulose solution via physical mixing and subsequent freeze-drying of the resulting suspension has major impact on the scaffolds performance in vitro and in vivo. Compared to HA-particles with of 20 µm in diameter, particles with 100 nm in diameter have exceeding properties regarding cell adhesion, proliferation, and expression of osteogenic markers in vitro using MG-63 osteoblast-like cells, as well as pronounced formation of newly mineralized tissue in a rabbit calvarial defect model (Figure 4) [127]. Cellulose and CDHA are used to fabricate a latticed scaffold via a 3D-printing process. By using an ethanol bath at the target site, a fibrous microstructure is established in contrast to smooth struts without ethanol. After optimization of the processing parameters, in vitro investigations using MC3T3-E1 mouse preosteoblast cells show no differences between the different microstructures for protein adsorption and cell proliferation but a significant increase in ALP activity and calcium mineralization for the scaffolds with the developed microstructure [131]. Incorporation of 1–4 wt.-% bacterial cellulose (BC) fibers of 100 µm length in a CPC results in enhanced mechanical properties and has an impact on MC3T3-E1 mouse embryonic osteoblast precursor cells. The best results for both the mechanical properties and biocompatibility are determined for composite materials containing 2 wt.-% BC. An even distribution of BC-fibers in this formulation is considered to cause these good results, whereas higher contents of the organic additive tend to agglomerate in the CMC [132]. Porous BC-sheets are prepared by laser perforation followed by NaIO4-oxidation to improve the biodegradability of BC and subsequent HA deposition using an alternate soaking process. Besides a well-defined structure consisting of trapezoid-shaped pores of 300 µm in diameter with a honeycomb arrangement in a nanofibrous BC-network (Figure 5), the described materials showed sufficient biocompatibility for human bone marrow-derived stem cells [128]. 

The alternate soaking process is also used to mineralize nonwoven sheets of CMC, resulting in a mixed CaP-phase of HA and brushite. Culturing with hMSC, non-mineralized scaffolds stimulated the early phase of differentiation as indicated by ALP-expression, whereas CaP-loaded scaffolds promoted expression of marker genes for later phases of osteoblast differentiation as well as calcium deposition in human mesenchymal stem cells. In a critical-sized mouse calvarial defect model, more new bone formation was observed for the mineralized CMC-sheets, whereas the unmineralized scaffolds had no effect compared to the untreated defect [133]. In a subsequent study using a dog lateral femoral condyle defect model, mineralized scaffolds loaded with basic fibroblast growth factor (bFGF) confirm the earlier findings with a higher new bone forming rate compared to control and unmineralized CMC-sheets. However, there was no significant difference between CaP-loaded scaffolds with or without bFGF. Thus, bone regenerating properties of the mineralized scaffolds are attributed to the gradual release of calcium and phosphate ions in the defect site [134]. 

As major components of the extracellular matrix, glycosaminoglycans (GAGs) like hyaluronic acid and chondroitinsulfate are a bioinspired class of scaffold materials providing biocompatibility and inherent bioactive properties (like enhanced cell viability) that are beneficial for bone regeneration [136]. However, lacking mechanical properties and having a high biodegradation demand, the need for composite materials for BTE in general and GAG-CaP hybrid systems in particular [137]. By adding 20 wt.-% hyaluronic acid to biphasic CaP (60/40 HA/TCP) granules, the occurrence of proinflammatory macrophages is significantly decreased in an in vivo rat cranial bone defect while the material’s biocompatibility is not affected [138]. Multichannel granules consisting of BCP are fabricated by a multipass extrusion method and subsequently suspended in a hyaluronic acid solution to yield an injectable material with improved biological properties. In vitro assays using MC3T3-E1 cells and an in vivo rabbit femoral condyle defect model proved the exceeding properties of the hybrid material regarding cell viability and proliferation as well as bone tissue growth together with the upregulation of osteogenic gene and protein expression [139].

Using EDC/NHS-chemistry, hyaluronic acid is derivatized with glycidylmethacrylate and thereby accessible for gelation via photocross-linking. By immersing the formed hydrogels in a Ca- and P-containing solution, followed by immersion in aqueous ammonia solution, different amounts of homogeneously distributed HA are formed in the hydrogel network depending on the concentration of the Ca/P-solution. Compared with the microscopic properties of a composite material obtained by simple physical mixing, the homogeneous dispersion of nanosized HA in the studied material results in improved mechanical properties as well as biocompatibility for L929 fibroblast cells and significantly reduced degradation rates in vitro and after 4 and 8 weeks in vivo testing on rats [137]. By additionally soaking the mineralized scaffolds in a solution containing different multivalent cations, the authors found that the incorporation of Ca^2+^, Ba^2+^ or Sr^2+^ is favorable to further enhance mechanical properties together with cell proliferation and ALP-activity of MC3T3-E1 preosteoblastic cells [140]. The effect of chondroitinsulfate on a CPC is evaluated by adding different amounts of the GAG to the phosphate-containing setting liquid before mixing it with the Ca-containing powders. Although maintaining the mineral phase, morphology, porosity, and compressive strength, the composite paste has improved injectability and anti-washout properties. Furthermore, the addition of chondroitinsulfate accelerated the adsorption of fibronectin what synergistically leads to significantly improved adhesion, proliferation and differentiation of mesenchymal stem cells on as shown by in vitro experiments [141]. 

#### 3.2.2. Miscellaneous Additives

Although the biological role of silicon is yet not understood in detail, in vitro and in vivo results evidence the beneficial role of Si on bone metabolism and homeostasis by promoting osteoblastic activity and inhibiting the activity of macrophages and osteoclasts [142]. Accordingly, the use of silica and silicate-based materials like bioactive glasses (BG) for BTE applications is investigated for decades. Like CaP-based materials, drawbacks like mechanical brittleness and lacking vascularization may be overcome by preparing hybrid materials together with polysaccharides. SiO_2_-nanoparticles are incorporated in a hydrogel fabricated by photocrosslinking of methacrylate- and methacrylamide-functionalized alginate and gelatin. Prepared hybrid materials exhibited cytocompatibility with MG-63 osteoblast like cells as well as mouse embryonic fibroblasts [143]. In a following study, the authors used genipin-cross-linked chitosan and collagen as matrix material for the incorporation of SiO_2_-NPs of 240 or 450 nm in diameter. In vitro assays proof the biocompatibility of the materials using hBMSCs and show enhanced ALP activity. Expression of marker genes reveals that hybrid materials with smaller SiO_2_-NPs promotes osteogenic differentiation of hBMSCs more effectively than materials with lager particles even in the absence of any osteoinductive medium [144]. Electrospun chitosan nanofibers have been applied to host mesoporous silica nanoparticles (MSN). Although the results for cell attachment and proliferation are comparable to native chitosan scaffolds, hybrid materials significantly promote the osteogenic differentiation of MC3T3-E1 osteoblast cells as indicated by measurements of ALP-activity and calcium deposition using alizarin red staining [145]. 

The known antibacterial properties of silver nanoparticles are a favorable feature for scaffolds used in BTE. Thus, in a recent publication AgNPs entrapped in a matrix comprising carboxylated cellulose gained via TEMPO oxidation of cellulose are used together with CMC and chitosan to prepare scaffolds by lyophilization. The highly porous scaffolds exhibited good protein adsorption and antibacterial activity against *E. coli* and *E. hirae*. Incorporation of AgNP improved mechanical strength and showed no negative influence on MG-63 cell compatibility tests [146]. Comparable results are found for AgNPs incorporated together with HA in an alginate-chitlac (lactose-derivatized chitosan) matrix, as the resulting microbeads show bactericidal effects against *S. aureus*, *P. aeruginosa*, and *S. epidermis*, while preserving biocompatibility towards osteoblast-like cells [147].

Including magnetic nanoparticles in a bone scaffold materiel offers the ability for diagnostic procedures as well as for stimuli responsive drug delivery applications. Composites of chitosan and magnetite NPs are combined with different biopolymers (hyaluronic acid, bovine serum albumin, gelatin) and subjected towards a biomimetic co-precipitation with CaP. The resulting materials exhibit a porous structure, are resistant towards biodegradation and have no cytotoxic effect on MC3T3-E1 preosteoblastic cells and thus are potential candidates for further studies in the field of BTE [148]. A magnetite-containing scaffold from silk fibroin and chitosan, cross-linked with glutaraldehyde, was prepared using freeze-casting. Cell culture and MTT assay on MG-63 osteosarcoma cells showed no adverse effects. Tunable porosity, water permeability, and good cell compatibility give reason to expect a potential use as bone scaffolds [149].

Being able to induce osteogenesis of stem cells and improving mechanical properties of polymer-based matrices, graphene and its derivatives like graphene oxide display an interesting alternative to established additives like CaPs in BTE [150]. Scaffolds comprised of chitosan, gelatin, TCP and graphene oxide are prepared via 3D-printing and subsequently treated with l-ascorbic acid to reduce graphene oxide and thus enhance mechanical and osteogenic properties. Besides enhanced ALP-activity of human osteoblast cells and Ca-deposition on the scaffolds, scaffolds showed antimicrobial activity towards *S. aureus* and *E. coli* without a negative effect on viability and proliferation of osteoblasts [151]. Chitosan-graphene oxide hybrid scaffolds have also been developed by directional freezing followed by lyophilization to create a porous and aligned 3D morphology. Seeding with MC3T3-E1 cells indicate that these cells attach and align parallel to the prepared channels [152]. Another group also developed chitosan-graphene oxide scaffolds via freeze-drying. In vitro ALP activity assay on 3T3-E1 murine preosteoblasts showed higher ALP levels on samples containing chitosan and 3% graphene oxide. These scaffolds were also tested in a critical bone defect model in mouse calvaria in vivo. Results show that they outperformed scaffolds with less or no graphene oxide after 72 h as well as 18 weeks after implantation. ALP expression levels increased and the percentage of newly formed bone measured via histomorphometry was significantly higher. Markers for osteogenic differentiation of bone cells were also investigated during the in vivo studies. Here, the early marker Runx2 was increased in the first 4 weeks and decreased afterwards. OPN and OCN levels, both late markers, gradually increased over the course of the 18 week study [153].

## 4. Future Perspectives

The interest in the regenerative potential of mesenchymal stem cells (MSCs) and their paracrine secretions has been noted in the recent years [154]. Moreover, the differentiation capacity of stem cells isolated from different sources provides a large range of opportunities for regenerative applications [154]. However, since many issues with cellular and acellular components of composite bone grafts remain unresolved, the search for the best cellular sources, combinations of osteoinductive molecules and scaffolds will continue in the upcoming years. Market analysis studies are assuring and predict an expansion of the market for artificial bone grafts accompanied by a distinct progress in clinically applicable technologies for preparation of stem cell-based customized bone grafts [155]. However, despite the huge amount of research in the field, only very small applications ever make it to clinical trials. Concerning the biomaterials, most clinical studies with respect to bone grafts use either pure calcium phosphates or hybrid materials based on collagen and calcium phosphates. For novel polysaccharide-based scaffolds to be successfully integrated into clinical studies, they must be at least equivalent or superior to existing systems in terms of biocompatibility, handling, cost, and bone healing. Other advantages of MSCs are the autologous origin and immunomodulatory properties. However, the main obstacle is the expansion of primary cells into large quantities, without compromising the quality and genomic stability. Large number of cells are required for identity verification, quality control, and expansion for grafting [156,157]. In addition, cellular heterogeneity and clonality have to be evaluated during scaled-up culturing [158]. It is anticipated that advances in cell sorting, expansion, and cultivation systems such as high-throughput bioreactors of various types [159] and closed adherent cell culture systems [157] will allow to overcome quality issues with time thus ensuring the safe use of MSCs in patients. This, of course, has to be accompanied by international regulatory framework adjustments for cell-based products manufacturing and use in trials and clinics [158]. Future effort will be aimed at establishing efficient and safe large-scale cell expansion protocols and harmonization of the regulatory issues related to clinical use of MSCs in regenerative medicine procedures such as tissue grafts. Chemists and material scientists working in the field of scaffold development will mainly be directed towards environmentally benign scaffolds. The desire is to produce highly advanced functional scaffolds by using renewable resources (terrestrial and marine biomass) with a combination of green chemistry aspects in material synthesis with highly advanced functionality of the target scaffolds [160]. Here, the challenge is quality control and assurance of the biomasses used for material development [154]. Additionally, future approaches should focus not only on what is technically possible, but even more on what is required and desired in order to develop a sustainable and functional scaffold.

## Figures and Tables

**Table 1:** Summary of calcium phosphates and their mixtures mentioned in this review.

Name	Abbreviation	Formula/Description	Ca/P Ratio
Hydroxyapatite	HA	Ca_10_(PO_4_)_6_(OH)_2_	1.67
Tricalciumphosphate	TCP	Ca_3_(PO_4_)_2_	1.50
Brushite	-	CaHPO_4_·2H_2_O	1.00
Calcium-deficient HA	CDHA	Ca_10-*x*_(HPO_4_)*_x_*(PO_4_)_6-*x*_(OH)_2-*x*_	<1.67
Whitlockite	WH	Ca_18_Mg_2_(HPO_4_)_2_(PO_4_)_12_	1.29
Biphasic calcium phosphate	BCP	Mixture of HA & TCP	-
Calcium phosphate cement	CPC	Mixture of multiple CaPs	-
